# Dementia Care: Intersecting Informal Family Care and Formal Care Systems

**DOI:** 10.1155/2014/486521

**Published:** 2014-02-20

**Authors:** Prabhjot Singh, Rafat Hussain, Adeel Khan, Lyn Irwin, Roslyn Foskey

**Affiliations:** ^1^Disability Services, Queensland Department of Communities, Child Safety and Disability Services, Brisbane, QLD 4001, Australia; ^2^University of New England, Armidale, NSW, Australia; ^3^School of Rural Medicine, University of New England, Armidale, NSW 2351, Australia; ^4^Aged Care Standards and Accreditation Agency Ltd., Sydney, NSW 2124, Australia

## Abstract

Dementia is one of the major causes of disability and dependence amongst older people and previous research has highlighted how the well-being of people with dementia is inherently connected to the quality of their relationships with their informal carers. In turn, these carers can experience significant levels of emotional stress and physical burden from the demands of caring for a family member with dementia, yet their uptake of formal services tends to be lower than in other conditions related to ageing. This paper is based on a qualitative study undertaken in the Australian state of Queensland and explores issues of access to and use of formal services in dementia care from the perspective of the informal family carers. It identifies three critical points at which changes in policy and practice in the formal care system could improve the capability of informal carers to continue to care for their family member with dementia: when symptoms first become apparent and a diagnosis is sought; when the condition of the person with dementia changes resulting in a change to their support needs; and when the burden of informal care being experienced by the carer is so great that some form of transition appears to be immanent in the care arrangement.

## 1. Introduction

Dementia as one of the major causes of disability and dependence amongst older people is an important aspect of formal service provision within aged care. Dementia is an umbrella term applied to more than one hundred degenerative brain syndromes which include Alzheimer disease, vascular dementia, dementia with Lewy bodies, and frontotemporal dementia [1]. These conditions are characterised by cognitive impairment and can result in communication difficulties, loss of memory, problems in performing previous routine tasks, and personality, mood, and behavior changes [1, 2].

The well-being of people with dementia has been linked to the quality of their relationships with their informal carers [3]. In spite of research, both in Australia and elsewhere in the world, identifying the significant levels of strain and burden informal carers often experience from the emotional stress and physical burden of caring for a person with dementia the uptake of formal in-home and community care services is lower among the carers of people with dementia than for other conditions associated with ageing [4, 5]. Canadian research suggests that among the explanations for the poor uptake of services is that the informal carers of people with dementia are either unaware of the services which are available or that these services are inaccessible, inconvenient, or too expensive [6]. There are also crucial differences in power, status, and authority between the informal and formal care system that need to be considered in the intersection of the two systems [6].

The policies and practices of the formal care system are conceptualized in rational and decontextualized terms, whereas the expectations and experiences of informal family carers emerge out of a unique set of personal circumstances. This suggests a need for medical and aged care services to consider not only the impact of caring for a person with dementia on their family, but also the family's perspective on formal services [2, 7–9].

The aim of the current paper is to explore the intersection of informal family caregiving and the formal care systems from the perspective of the primary family carers of people with dementia in an Australian context. The study focuses on issues of access to and use of formal services in dementia care from the perspective of the informal family carers. Recent research has found that the decisions made by informal carers on whether to seek or reject formal service provision arise through a complex interplay of social, moral, emotional, and cultural issues [10]. Thus, negotiating the intersection between family carers and those involved in designing and delivering formal aged care services involves social actors who belong to different systems each of which has specific configurations and ideologies [11].

## 2. Background to the Formal Aged Care System in Australia

In Australia, each year, more than million older people (out of a total population of just under 23 million) receive some form of formal support through the aged care system [12]. Although older Australians prefer home-based care to residential care, the greatest proportion of government funding for aged care services continues to be spent on residential aged care services [13]. In June 2010 the distribution of government subsidized aged care places for people aged 70 years and over within the state of Queensland (the state where the study reported in this paper was undertaken) was 95.6 per thousand in residential care, 3.9 per thousand in high level community care, and 20.8 per thousand in low level community care [12].

The service provision responsibilities are split both at the level of funding and frontline services for people who are ageing. The Australian service system has two streams of community service provision; home and community care services are jointly funded by the national and state governments, whilst home care packages are fully funded by the national government. Within Australia, not only the national and state levels of government do provide two different forms of community and dementia care and support, but also the actual provision of services then occurs through a combination of nonprofit and for-profit providers, which, in turn, may be managed at a local, regional, and state level. As a result in the local area there can be multiple providers with whom the carer comes into contact, in some cases providing the same or similar services. This causes considerable confusion for carers due to the complicated and overlapping formal service system. At the frontline of service delivery this is often perceived to have negative impacts due to competition between providers, along with issues related to internal organizational dynamics and interorganizational relationships.

In Australia there are economic benefits to government in supporting informal carers to continue to care for people with dementia as long as possible, thus reducing admissions to residential care services. In 2010 across Australia the cost of residential aged care provision was on average $48,710 per person per year, with government bearing 69% of this cost (or $33,610); in contrast the average annual cost of providing a person with in-home formal aged care was $7,520, with government bearing 92% of the cost (or $6,918 per year); the cost of mixed informal and formal in-home care was on the average of $11,370, with government bearing 35% of the cost of care (or $3,983 per year); and in-home informal care with the person receiving no support from formal aged care services was estimated to cost $10,880 per year, with government bearing no direct cost [13].

## 3. Methodology

The research reported here was undertaken in the Australian state of Queensland using a qualitative research design incorporating descriptive thematic analysis. This method was chosen as was congruent with the research aims, allowed for an in-depth understanding of the informal caring experience of family carers of people with dementia, and enabled the experience of carers to be presented from their own point of view.

Seventeen family carers involved were recruited from across Queensland, the third most populous state in Australia with a population of 4.6 million people. Selection of participants included in the study was based on the person's experience of caring for a family member with dementia living at home, irrespective of the duration, frequency, or status of the care relationship. Those recruited came from throughout southeast Queensland including metropolitan Brisbane (the third most populous city in Australia), regional centres, and rural communities.

All participants were volunteers and recruited through advertisements in local papers, along with distribution of information through two key nongovernment bodies in the state: the Alzheimer's Association and Carers Queensland. Eight of the interviews were conducted face to face and nine via were conducted the telephone—including all of those with participants living in rural and remote areas of the geographically large state.

The sample included two males and fifteen females, a gender balance consistent with the distribution of primary carers of people with dementia in Australia. In the study carers were categorized into two groups based on the age of eligibility for the Australian Aged Pension—as younger carers where they were under the age of 65 years and as older carers when they were aged 65 years and over at the time of the research interview.

The sample included

- two sons both of whom were aged less than 65 years;
- five daughters, all aged less than 65 years;
- one daughter-in-law aged less than 65 years;
- nine wives, of whom two were under the age of 65 years and seven aged 65 years and over.

## 4. Data Analysis

The individual interviews were audio-recorded, with prior consent and transcribed verbatim. The transcripts were read several times to develop a coding scheme based on thematic analysis of the data [14]. To improve audibility, other members of the research team also reviewed the transcripts and the emergent themes. The first author, who conducted all the interviews, brought to the experience both her background on dementia care as well as on policy and practice development in aged care. That is, within the research process neither the researcher nor the person being interviewed came to the interview “untouched” [15] by the experience of caring for a person with dementia, albeit from very different perspectives. Other members of the project team provided their own perspective from their work in health services management and sociology of health. Rigour was gained through incorporating three key elements—validity, reliability, and verification related to sampling, the position of the researchers, and assessment of whether the objectives of the research were met—into the analysis process [16].

The analysis included a constant comparison method with roots in grounded theory along with descriptive thematic analysis, an inductive, or data driven, method that began with the development of a coding matrix based on the questions used in the interviews. The themes were named, defined, described, and refined though a six-stage process which allowed the analysis to become richer in explanation as the process progressed [14, 15]. A subsampling technique, used as a comparative technique, was used to draw out codes by comparing each participant's experience, considered unique in its own right [17]. The clustering of thematic codes to develop higher order themes was a crucial step in the identification of patterns connected to the broader questions under investigation [18]. These higher order themes were then arranged into a conceptual clustering matrix to allow conclusions to be drawn from the data [19]. Through this rigorous process the key findings of the research were schematized to answer the question of how family carers of people with dementia living in the community were successfully managing, continuing, and sustaining informal caring in the present and how they would continue to care in the future. The coding schema was reviewed and corroborated over several face-to-face discussions by three senior academic researchers, who are also coauthors of this paper.

## 5. Results

The uncertain and unpredictable nature of caring for a family member with dementia was one of the key concerns of the carers, influencing their ability to manage and continue with their caring situation. The carers described the total change in their lives as a result of their caring responsibilities and expressed their desire to continue caring for their family member with dementia but noted that in order to do so they needed access to formal services capable of providing more flexible support that recognized the concerns and issues experienced by carers. From the carer's perspective recognition of the importance of their role was about ensuring timely provision of information and access to formal care early in their caring journey, rather than access to formal care only as a crisis response when their situation deteriorated.

The two major themes which emerged were related to the challenges they had experienced in their interaction with medical and aged care services and enablers and impediments at the level of formal community support services. The analysis revealed several distinct subthemes embedded within both the major themes. In relation to interaction with medical and aged care services the sub-themes included delays in the initial diagnosis of dementia, barriers experienced in seeking access services, and a lack of understanding of the needs of informal carers of people with dementia by service providers (see Table 1). For enablers and impediments at the level of formal community support services, the participants identified two key characteristics of formal community services which would assist in their ability to balance the effort of caring and their own capacity to continue to care: quality of in-home and day care services and appropriate and accessible opportunities for carer participation in policy formulation and training of staff employed by the aged care and community-based formal services (see Table 1).

### 5.1. Challenges in Carer Interaction with Medical and Aged Care Services

The family carers described the challenges they had experienced in gaining the initial diagnosis and accessing appropriate medical treatment for their family member with dementia, including timely access to appropriate specialist care. Several carers suggested that the local general practitioners familiarity with their family member interfered with the objectivity of the medical assessment impeding early diagnosis. Two of the carers explained in some detail how they felt it was their family member's past medical history, of posttraumatic stress disorder in one case and mental illness in another, which had contributed to the delay in diagnosis. In the first case it had taken seven years from the time the first symptoms of dementia became apparent and were drawn to the attention of the local general practitioner to the eventual diagnosis of dementia. As the wife/carer explained *seven years is a long time *to wait for a diagnosis. In the second case there had been a three-year delay in diagnosis, resulting in lost opportunities in medical treatment. As that man's wife/carer explained, *“You need to be on them *(referring to drug treatment) *early. So, not much use waiting for the doctor to decide that there is a problem, after three years of, of saying it's depression” *(wife carer).

Not only some of the family careers had experienced delays in diagnosis and referral to other medical services but also this had then been exacerbated, in several cases, by a poor quality response by the medical professional at the time of the disclosure of the diagnosis. Carers described doctors who failed to take the time to listen or respond to the queries which were raised in ways they felt as insensitive to the needs of both the person with dementia and their own needs as the person's primary carer.

The carers also identified the impediments they had experienced in referral to specialist medical services in dementia care. Regardless of where they lived the family carers identified timely and appropriate access to specialist services as a crucial element in their interaction with and confidence of the formal service system.

One carer described the delays she and her mother had experienced in accessing appropriate services in terms of age discrimination embedded within the health care system as follows:

*And so now a geriatrician is taking care of her but such a long-winded process that should have been so much easier. Had it seemed to us that she mattered, but she just didn't seem to matter. So my feeling was well perhaps when you're over 65 or—and/or experiencing dementia, that you just drop to the bottom of the pile, I find that incredibly offensive.*

Several family carers described how they had been left feeling stressed and demoralized when the medical specialist failed to take the time to properly listen to and adequately respond to their questions and concerns, around the implications of changes in the person's behaviour and capabilities. The following description by a wife/carer is indicative of the absence of explanation, advice and support by some medical specialists, “*the doctor just said, just go home and live out the rest of your life as well as you can.”* “*That's it. I wasn't told anything. I wasn't given any advice, nu-nu-nothing!”*

A specialist's dismissal of the carers concerns, can also mean a misdiagnosis as in this description by a daughter/carer:

*So she was sent to a geriatrician at the Base Hospital. He was quite good but a little bit arrogant, a little bit condescending, a little bit smug, dismissed dementia and said, no it wasn't dementia, it certainly wasn't Alzheimer's and he thought it was probably MS. *

This misdiagnosis added to the challenges being experienced by this family carer, as the care recipient continued to display symptoms associated with dementia, yet the misdiagnosis meant that the daughter was unable to access appropriate dementia-specific services and support. Fortunately not all medical practitioners consulted by those involved in the current study had acted in this dismissive way, with several of the carers describing medical practitioners who had been very helpful, ensuring both their family member with dementia, and they as the primary carer, understand the medical implications of the dementia diagnosis.

### 5.2. Lack of Information on How to Seek Nonmedial Support Services

Almost all the carers described challenges they had experienced in getting appropriate and timely information about the nonmedical support services available to people with dementia and their carers. Initially this was indicative of the poor intersection between medical and non-medical services in aged care. As one wifecarer explained, *my GP is very nice and very good, but he didn't mention where to go or how to access it* (non-medical information and support). *Because (medical practitioner) is the first port of call, if even if he just had a paper with a code on it that this is Alzheimer's society or whatever name, give them a call, that was all that was needed.* The carers believed that information on support services was of equal importance as medical assistance in the diagnosis of dementia. Overall what emerges from the interview data was the importance of the doctor's approach in supporting the family carer's capacity to manage the caring situation, especially in planning for future care needs.

As explained by one wife/carer the compounding impact of providing informal care is invidious,

*because you feel duty-bound to keep going, and I think because you've eased into it month by month, year by year you just eased in and you're doing more and more and more and you don't know.*

A number of carers described their frustration that even where they had been provided with information on services in their local area early in their journey as carers this had often been *out of date*. Carers had also experienced different agencies *duplicating the same information, resulting in overlap and confusion*, yet still leaving them with gaps in their understanding of the extent of the options potentially available through the formal aged care system.

Carers also described the confusion resulting from a dual stream of community service provision between national, state, and local systems which results in multiple providers with which the carer comes into contact, in some cases providing similar or the same type of services. Rather than perceiving the choice of providers as positive, the carers felt that a lot of resources were being wasted on managing an unnecessarily complicated and overlapping service system. Several carers highlighted the negative impacts of competition between providers, along with issues related to internal organizational dynamics and interorganizational relationships.

In the present study those family carers with previous experience in engaging with the health and community sectors found that being familiar with the ways support services operated advantaged them in negotiating at the intersection of formal and informal care and support. The lack of familiarity with the organizational logic and work routines that underlies such interactions served to heighten family carers sense that they have no control over the situation they have found themselves in, at a time when they are already stressed and vulnerable. To add to the confusion they found that a single term, such as respite care, was often being applied to differing forms of service provision. For example, in relation to respite care, the same term is used for in-home care, a centre providing day respite, and a block of respite care in a residential facility.

Carers also highlighted their difficulties in accessing services soon after diagnosis, with several carers indicating that the services they had contacted appeared to give little priority to the needs of people in the early stages of dementia. In rural areas, in particular, several carers were given the choice of either accessing generic services unsuitable for the needs of their family member with dementia or dementia-specific services which were inappropriate as they were designed around the needs of those with more severe symptoms and behaviours. Other carers highlighted their frustration when they found that the generic community aged care services they had contacted effectively denied access to people with a diagnosis of dementia. Delays experienced in access to some services were also a concern for the carers with long waiting periods not only causing frustration, but also leaving the carer feeling that no one cared for them, whilst they cared for their family member with dementia.

Carers described coming into contact with a community aged care system which they felt had become a business, rather than service, a system which cared more about managing finances than meeting the complex needs of people with dementia, along with those of their informal carers. The commodification of services is paradoxical for it also raises carers expectations in relation to the availability of case management as illustrated by the following comment by a daughter/carer:

*I also believe that if they're providing a seven-day service then there needs to be a contact number for them for all seven days because you can't actually ring them on a Saturday morning and say what's happened because there's nobody there. There's no one to answer the phones.*

### 5.3. Lack of Understanding of Needs of Informal Carers by Formal Care Services

A crucial factor that encouraged carers to become actively engaged with formal support services was care workers who were proactive in their contact, rather than relying on the carer to initiate all the interaction with the service provider. In these circumstances the service was more likely to be perceived by the carer as complementing, rather than undermining, their capacity to sustain their caring role. Carers expressed particular concerns about carers *effectively having to do their own case management*, with the pressure always being on them to initiate contact with services.

However, most often the assessment and monitoring processes being used by the formal services were described by the family carers as inflexible and the services as lacking in the ability to respond in a timely way to the constantly changing demands and emergent needs involved in caring for a person with dementia. A failure by assessors in the formal system to adequately consider the needs of the carer is evident in the following description by a daughter/carer:

*We will only give you X number of minutes in service, we will only do this and we will only do that and it will cost you part of your income and it was just, I thought I honestly suspect that part of this was not meant to be easy to access.*

This is indicative of the different logics underlying formal and informal care systems. The carers in the present study believed that their active involvement in the decision-making process would allow for the best interests of the person with dementia to remain paramount in formal care provision. They explained how it was through their involvement in decisions relating to formal services that they were able to develop their confidence and trust in the system increasing the possibility that they would continue to access services. Conversely a complex assessment process, which left carers feeling excluded or peripheral, was identified as an impediment to constructing a comfortable and trusting relationship with service providers.

Whilst some carers noted that their role was nominally recognized by services, this was most often only a passive recipient of support, rather than an active participant in the support and care process. The carers emphasized that the formal community aged care system needs to be rebalanced and refocused to ensure that carers are conceived and included as equal partners at the intersection of formal and informal care systems. Carers suggested that this would assist formal care providers to be more responsive and accountable as reflected in the following comment by a daughter/carer:

*I'd like the care providers to be more accountable to carers and have to explain why they do the things that they do and not just treat their clients like it's a bloody car service or whatever. I think they should be accountable to treat them as people and they just can't have one rule for everybody.*

Carers considered the availability of services to complement their role as informal carers as critical to the future sustainability of their informal care. With many formal services restricted to week-day provision the carers highlighted that services needed to be responsive to the continuous nature of caring by broadening their coverage to seven days a week. The impact of service rationing on the carers own sense of wellbeing and social engagement can be significant as this son/carer explained, *I get four hours a month off, which isn't much time to socialize*…. “*I haven't got a social life any more*.” Other service-related issues experienced by carers included barriers to the transportability of care and support arrangements across the structural boundaries of local, nongovernment, state-based, and national service providers and systems.

In some situations, the carers had found that there was no appropriately flexible and responsive service available. This included those carers who still remained in the workforce and those who would have liked the option to continue working alongside their caring role, but had found it impossible as in the following description by a daughter carer:

*I had no one to look after mum, so I couldn't go to work, and I do believe that that impacted and I do believe that that's one of the reasons that they fired me. Because I couldn't attend work because I had to look after mum.*

### 5.4. Quality of In-Home and Day Carer Services

A number of those receiving formal in-home services in the Queensland study described how they felt a need to monitor services when workers unknown to them came into their home. Some carers expressed particular concerns about the quality of in-home services. They described circumstances in which in-home respite care assistants were constantly changing, did not turn up when expected, did not demonstrate compassion towards the person with dementia, and did not have good communication skills or demonstrate understanding of the care needs of both the carer and their care recipient. Such issues contributed to the carer's levels of worry and stress as they believed that the vulnerability of the person with dementia and their lack of familiarity with the in-home worker were a risk that needed to be actively managed. The carers described a number of strategies they had used to handle the risks they perceived in using in-home services: staying around the home whilst care was being provided, familiarising themselves with the care system, and most importantly accessing only those services they felt to be reliable and trustworthy.

On the same vein the quality of day care services was perceived to be problematic. The poor physical and interpersonal environment within some out-of-home services encountered by carers deterred them from accessing that service for their family member, particularly when they knew that their family member would not approve the facility. For example, this carer highlights a need to improve both the physical infrastructure of day-care facilities and the education of staff, “*it is not the facility it is just a chair, and that is hard to send someone to a place for a day where they just sit in a chair*.” Central to the carers concern was the fact that this situation potentially compromised the safety and security of the person with dementia. In all types of out-of-home environments the carers sought a home-like quality and their ideal was for sufficient respite options to be available so that the most appropriate alternative could be selected, which met the particular needs of their family member with dementia.

Creating a trusting relationship with both residential and in-home respite services was factor which had helped the carers feel more confident about dealing with emergencies and sudden changes to care arrangements. Almost half of the carers described the vital role of respite care in allowing them to attend regular commitments which included, but were not limited to, paid work and carer-related support activities (e.g., attending support groups and committee meetings). Respite, in these terms, was not necessarily providing the carer with a break from engagement in instrumental tasks, just time away from the care recipient to complete necessary chores or to earn a living. The carers expressed a need for respite options to be more widely available. They also identified a need for services to be responsive to the needs of carers recovering from personal crises, including those that were health-related.

It was through consistent, good quality, service provision that the trust and confidence of the carer in formal care arrangements gradually increase. The carers considered consistency in workers providing good quality care and support to be fundamental in meeting the needs of the care recipient and through this in also supporting them to sustain their informal care.

### 5.5. Appropriate and Accessible Opportunities for Carer Participation in Formal Services

Almost half of the carers interviewed already played an active role in providing support for other carers, reflecting the recruitment process for interview participants through the Alzheimer's Association and carers organizations. Despite this carers also highlighted barriers in participation in consultation processes such as carer forums and carer support groups, including issues related to the timing and location of such events, as in the following comments of this daughter/carer:

*I got an email the other day and they're actually having a forum in one of the big cities. This is the first time I've ever been asked but the thing is that I think it's at 10:30 in the morning, do you know what I mean? *

Several of the carers highlighted the vital importance of carer input into policy and planning and implementation of formal service provision. These carers felt they had inside knowledge from their caring experience that would be extremely valuable to improving formal services, as expressed by this daughter/carer, “*it doesn't matter how many university degrees and that people do, they don't know until they're a carer.” *…* What it really involves*.

These carers indicated that a greater investment in making support and training available to ex-carers to provide peer support would bring systemic benefits, including better carer and care-recipient outcomes. They suggested that combining professional expertise and peer support would assist the carers of people with dementia to work through their specific, unique, sensitive, and complex issues. Peer support was identified as important both in helping primary carers adjust to their changing circumstances and in assisting other family members to better understand the impact of dementia on the primary carer. Several carers indicated that they would be willing to contribute to the education or training of formal dementia care workers through sharing their own experience.

These carers also expressed concern about the shortage of appropriately trained aged care workers. They also highlighted the staff retention issues within community care services, due to the poor pay and conditions of frontline workers in formal community aged care, as in the following description by a carer/daughter:

*Well to tell you the absolute truth I think that they need to pay them more money. The actual people that come to the door and help these people are on the most terrible wage imaginable.*

These carers noted how the complex design and implementation of the service delivery system placed additional stress and strain not only on themselves as informal carers but also on frontline workers. At the interface of the two systems the frontline carer and the informal family carer come into contact within a social contract of providing care to the person with dementia in a form which exceeds the reciprocity which usually exists between adults, and therefore the continuity in workers allows for these interrelationships to develop and deepen which can be crucial to the sustainability of informal care.

## 6. Discussion 

The present study, along with the other recent research focused on the carers experience in dementia care, indicates that the quality of interaction is key to whether the relationships established add to or reduce the burden of care on family carers. There are three key points where changes to policy and practice in the formal care system could improve the capability of informal carers to continue to care. The first occurs when the early symptoms of dementia in the care recipient become apparent and lead to initial contact of the carer with the medical system. At this point information is very much a two-way flow between the informal carer and the formal community care and support system. It needs to be recognized that family members have historical information about the person that, if shared in a timely manner, has the potential to benefit everyone involved [20].

Previous studies both in Australia and elsewhere in the western world have found that the general practitioner's role is often vital, not only in the early detection of dementia, but also in the timely referral of the person to specialist services [21–23]. The Australian Institute for Health and Welfare [1] highlighted in their recent report on dementia care in Australia that as there is not a “single or simple test that will definitively diagnose dementia” the general aim is for the medical assessment process to gather sufficient information about “changed behaviours, functional capacity, psychosocial issues, and relevant medical conditions to allow a diagnosis to be made.” However, a recent survey of the dementia-related health literacy in the Australian and English general practice found that only 22 percent of general practitioners and 15 percent of practice nurses considered their dementia knowledge to be adequate [24].

It has previously been argued that when medical professionals are discussing dementia as a condition issues of concern must be considered from the perspective of both the person with dementia and the primary carer [25]. As mentioned earlier, the hierarchical organization of healthcare services and the esteemed status of health professionals particularly doctors as primary holders of knowledge leads to asymmetrical power relations in clinical encounters. Carers often feel disempowered as they have limited voice in relation to information on services and care on offer for their family member with dementia. There are also acknowledged structural constraints within the Australian medical system, with recent research finding that particularly in regional Australia general practitioners may delay referring a patient to a specialist for further testing, in part because of more limited access to specialist services [21].

The second critical point occurs when there is a change in support needs as the condition of the person with dementia changes, such as increasing concerns by the carer about the safety of their family member or the emergence of challenging behaviours. This is the point at which access to appropriate respite care may be crucial to the sustainability of informal care. Various approaches that would improve carers engagement with formal community care services, such shifting the focus of formal case management away from making referrals and providing a linking service between services, to a focus by the case manager on organizing personalised and flexible services which not only considered the present issues but also anticipated the future challenges for the carer. This includes consideration of the impact of the demands of caring the lives of people caring for a family member with dementia beyond their caring role to ensure that carers continue to have opportunities for economic and social engagement.

A recent inquiry by the Australian Human Rights and Equal Opportunity Commission highlighted the challenges faced by Australians seeking to combine work and informal caring responsibilities and identified serious implications for the emotional and economic wellbeing of carers over the long term [26, 27]. In the present study, the stress being experienced by the carers was identified by them as risking their ability to continue to fulfill their informal caring role alongside other responsibilities in their lives. It is important to note that some of the younger carers were combining their role as a primary carer with other family and work commitments, whilst several of the older carers interviewed in the present study did not have access to practical hands-on informal support on which they could readily draw in a crisis.

Health economists have conceptualized the burden of informal care in dementia as the putative cost of the time spent by the informal carer on the tasks of care. In their recent meta-review on informal dementia care Costa and colleagues [2] assessed the objective burden of informal care in two common dementia-related conditions: firstly Alzheimer disease at 55.73 hours per week and secondly Parkinsons disease at 15.8 hours per week. They then linked this objective measurement to the subjective evaluation of caregiver burden or strain noting that “the more the number of informal hours increases, the more the subjective burden is important” [2]. This is most evident where the person with dementia is exhibiting emotional difficulties and/or challenging behaviours [9, 26–28].

The assessment of the subjective burden of informal care by formal services needs to better understand the contextual, psychological, and emotional dimensions of care from the perspective of the carer [2]. The gap between apparent need and actual uptake of services points to an important mismatch between the logics operating within organizational systems and the logics within the family environments in which most of dementia care occurs [5]. For example, whilst formal systems tend to base their measurement of need around an abstract and lineal conceptualization of time, for the person with dementia and their informal carer (as well as some frontline staff) the framing of time is enmeshed with social relations and involves processes which cannot be hurried [29].

Other Australian researchers have highlighted that the gain that carers experience in receiving formal services is inherently ambiguous, for whilst formal services are providing support to family carers, they can also be undermining their sense of identity and control over their circumstances [5]. A number of those receiving formal in-home services in the present study described how they felt a need to monitor services when workers unknown to them came into their home. In their Tasmanian study Lloyd and Stirling [5] similarly noted that whilst access to formal services can offer physical, psychological, and emotional relief to informal carers it can also cause the carer to experience a threatening loss of mastery over the material and symbolic boundaries of previously private spaces.

The third critical point occurs when the burden of informal care is being experienced by the carer as so great that some form of transition is imminent in the care arrangement. As in the other research (e.g., [3, 6, 13]) the carers in the present study were committed to caring for their family member in the community as long as possible, yet also being very aware of the fact that there were limits in their capability to sustain their caring role, particularly as dementia (dependent on the condition) can be associated with progressive decline. They wished to begin to prepare in advance for the point was reached in which the person could no longer be cared for at home.

## 7. Conclusion

The issues which emerged through the present study, combined with other recent research in Australia, suggests that for informal dementia care to become more sustainable it is necessary to ensure that formal service provision is flexible enough to respond to the complexity of the circumstances (family, social, and economic), not only for the person with dementia but also for their informal carer/s. There is increasing recognition within Australian aged care funding and policy that the care and support of people with dementia is complex and needs to take the particular needs of carers into consideration. However, on the ground carers continue to be faced with confusing funding arrangements and a complex array of services provided by for-profit as well as non-profit organisations.

The present study has reinforced the findings of other reports [3, 26, 27] that in order to improve the sustainability of informal care of people with dementia in the community the formal community care system needs to take a more proactive approach in engaging with informal carers. In order to reduce the burden of care on the primary carers of people with dementia there is a need to ensure the carers perspective is being sought both in the development of policy and within everyday practice. Carers have noted that better services, including respite care, medical (professional and primary) services, and more responsive and appropriate information, and flexible and responsive services are the key considerations in the sustainability of informal care.

## Figures and Tables

**Table 1:** Main themes and subthemes based on carers perspectives of intersection of formal and informal dementia care services.

Main theme	Subthemes
Interaction with medical and aged care services	(i) Delays in initial diagnosis
	(ii) Lack of information for nonmedical support services
	(iii) Lack of understanding of needs of informal carers

Impediments and enablers at the level of formal community services	(i) Quality of in-home and day care services
	(ii) Appropriate and accessible opportunities for carer participation in formal services
